# A comparison of the burden of knee osteoarthritis attributable to high body mass index in China and globally from 1990 to 2019

**DOI:** 10.3389/fmed.2023.1200294

**Published:** 2023-08-23

**Authors:** Min Song, Huijing Chen, Jingyi Li, Weichang Han, Wenfeng Wu, Gaoyi Wu, Anqi Zhao, Qing Yuan, Jiani Yu

**Affiliations:** ^1^Medical College of Acu-Moxi and Rehabilitation, Guangzhou University of Chinese Medicine, Guangzhou, Guangdong, China; ^2^School of Public Health and Management, Guangzhou University of Chinese Medicine, Guangzhou, Guangdong, China; ^3^The Second Clinical College, Guangzhou University of Chinese Medicine, Guangzhou, Guangdong, China; ^4^Huatuo Hospital, Zhaoqing, Guangdong, China; ^5^The Second Affiliated Hospital of Guangzhou University of Chinese Medicine，Guangdong Provincial Hospital of Chinese Medicine, Guangzhou University of Chinese Medicine, Guangzhou, Guangdong, China; ^6^Sun Yat-Sen Memorial Hospital, Sun Yat-Sen University, Guangzhou, Guangdong, China

**Keywords:** knee osteoarthritis, high body mass index, epidemiology, Joinpoint, age-period-cohort model

## Abstract

**Background:**

Excess body mass index (BMI) plays a key role in the onset and progression of knee osteoarthritis (knee OA). However, the burden of knee OA attributable to high BMI at the global, Chinese, and regional levels have received far too little attention. The aim of this study is to provide evidence to support the design of policy by investigating long-term trends of years lived with disability (YLDs) for knee OA.

**Methods:**

To illustrate the trends of YLDs for knee OA attributable to high BMI and the temporal trends of the YLDs rate by age, period, and cohort, Joinpoint regression software and age-period-cohort (APC) were used to analyze the YLDs data of knee OA from the Global Burden of Disease (GBD) 2019.

**Results:**

In China, there were 549,963.5 YLDs for knee OA attributable to high BMI in 2019, which had increased by 460.7% since 1990. From 1990 to 2019, age-standardized disability-adjusted life year rate (ASDR) of knee OA attributable to high BMI trended upwards. The average annual percent change (AAPC) of knee OA attributable to high BMI in China and globe were 3.019, 1.419%, respectively. The longitudinal age curve of the APC model showed that the YLDs rates of knee OA due to high BMI increased with age, and YLDs rates were higher among females than males. The period rate ratios (RRs) of knee OA due to high BMI increased significantly. The cohort RRs of knee OA due to high BMI increased among those born between 1900 and 1970. The net drifts of knee OA attributable to high BMI in China and globe were above 1. Compared with global condition, the net drift values of knee OA attributable to high BMI in China was higher. Compared with females, males had higher net drift value. Countries with high socio-demographic index (SDI) have a much higher burden of knee OA caused by high BMI than countries with low SDI.

**Conclusion:**

In China, high BMI is a substantial cause of knee OA, the incidence of which has been increasing since 1990. In addition, women and the elderly are more vulnerable to knee OA caused by high BMI. The Chinese government must take the long-term impact of high BMI on knee OA into account and implement effective public health policies and resort to interventions to reduce the burden as soon as possible.

## Introduction

Osteoarthritis (OA) is one of the most common orthopedic conditions caused by aging which involves structural changes in affected joints, including cartilage degradation, synovial inflammation and bursa ligaments inflammation (1). Not only does it cause a heavy health burden, but it also puts a strain on the healthcare system (2). OA may result in disability eventually with its incidence and prevalence increasing year by year in the general population. People living with hip and knee osteoarthritis (knee OA) all over the world increased to approximately 300 million in 2019 (3). Worldwide, hip and knee OA ranks 11th among factors that cause disability. This disabling effect is primarily measured by years lived with disability (YLDs) (4). It is important to note that knee OA is the most common form of OA because knee is the largest joint in the human body (5–7). In addition, knee OA can lead to joint replacements in late stages (8–10). With China’s population aging, knee OA will be one of the leading causes of disability and societal cost among the elderly (11). According to Global Burden of Disease (GBD) 2019, the incidence of knee OA in China reached 84.258/100,000 in 2019, increasing by 128.7% compared with 1990 (12). There are increasing concerns about knee OA (11). Researchers found that age, gender, and obesity are independent risk factors of knee OA. Among them, knee OA is strongly associated with the increase of BMI (13), and the obesity increased the risk of OA by 3 folds (14–16). A meta-analysis (17) also showed a 5-unit increase in body BMI was associated with a 35% increased risk of knee OA.

Worldwide, overweight or obesity has become a serious public health problem. In 2015, 603.7 million people were obese (18). Overweight is defined as the value of body mass (BMI) is between 25 kg/m^2^ and 29.9 kg/m^2^, while obesity is defined as BMI exceeding 30 kg/m^2^. A high BMI is defined as its value ≥25 kg/m^2^ (19). The impact of the knee OA is primarily measured by YLDs and disability-adjusted life years (DALYs). Among the 15 most common causes of DALYs from 2010 to 2019 globally, high BMI stands out and it has the highest change rate (20). Globally, the YLDs associated with high BMI increased significantly from 1990 to 2017 (21). China’s obesity rate ranked 60th in 1975 worldwide, and jumped to 2nd in 2014 (22).

Several studies (15, 16, 23, 24) explored the association between knee OA burden and high BMI. However, there are few studies examining the burden of knee OA caused by high BMI in China, nor there are studies comparing burden of knee OA among China, global and regional levels. Furthermore, its association with age, gender, and sociodemographic index in China has not been illustrated. To address these limitations, up-to-date datasets were systematically collected from GBD 2019 to estimate the average annual percent change (AAPC) of YLDs for knee OA attributable to high BMI and age-period-cohort (APC) model was used to investigate the effects of high BMI on knee OA. Thus, this study is expected to provide support for evidence-based prevention programs in China in terms of disease burden of knee OA caused by high BMI.

## Materials and methods

### Data collection

The YLDs rate and number of patients diagnosed with knee OA attributable to high BMI were extracted from the 2019 GBD study database via Global Health Data Exchange (GHDx) query tool.^1^ To date, the GBD 2019 collected data on the burden of 369 diseases and injuries (including incidence, prevalence, mortality, YLDs and DALYs) and 87 risk factors in 204 countries and territories from 1990 to 2019. The current study examined YLDs for Knee OA attributable to high BMI and the age-standardized disability-adjusted life year rate (ASDR) per 100,000 attributable to high BMI in global and Chinese populations between 1990 and 2019. Gender and age of the patients and Socio-demographic index (SDI) values were collected to assess how they affected YLDs and ASDR. The YLDs represent how many years one lived with disability. SDI measures a regional development status by measuring lag-distributed income *per capita* and mean educational level for people aged 15 and older, and total fertility rate under 25 years (5). Based on the SDI quintiles, 204 countries and territories were categorized into five groups: high SDI (>0.805129), high-middle SDI (0.689504–0.805129), middle SDI (0.607679–0.689504), low-middle SDI (0.454743–0.607679), and low SDI (≤0.454743) (25). Previous studies reported detailed method about estimating the burden of diseases associated with GBD 2019 (26, 27). The institutional ethics committee exempted this study because data from the 2019 GBD are publicly available (28).

### Statistical analysis

From 1990 to 2019, Estimated annual percentage change (EAPC), which measures the age-standardized rate trend over a specific period (29), was used to evaluate the trend in ASDR attributable to high BMI. A linear regression model was used to calculate EAPC as follows (30):

ln(ASDR)=α+βx+εEAPC=100×(exp(β)−1)

In the equation above, x is the calendar year, ε is the error term, and β describes the positive or negative age-standardized rate trend. EAPC and its 95% confidential interval (CI) can be obtained from the model above. An increasing trend is considered as EAPC and its 95% CI both >0; a declining trend is considered as both EAPC and its 95% CI < 0. Otherwise, the burden of knee OA due to high BMI is regarded as stable.

A comparison of the percentage change between 1990 and 2019 can be found on the IHME website.^2^ Using DisMod-MR 2.1, a Bayesian meta-regression tool, GBD estimates the number of quantifications more than 1,000 times, with 95% UIs (uncertainty intervals) determined by the 25th and 975th values of the ordered 1,000 estimates (21). Moreover, Spearman’s rank test was used to determine whether high BMI related knee OA burden (ASDR) correlated with SDI. In addition, the YLDs for knee OA were decomposed by age structure, population growth, and epidemiologic changes through decomposition methodology of Das Gupta (31), and R soft 4.2.1 was used for statistical analysis and chart visualization.

### Joinpoint regression analysis

The AAPC and 95% CIs (confidence intervals) were calculated using JoinPoint software (Version 4.9.1.0) and were used to analyze trends over 30 years of YLDs attributable to high BMI. JoinPoint software was used to estimate mortality data, which using a grid search method and Monte Carlo permutation tests to optimize the model. Suppose there is a sequence of observations (x1, y1) …, (xn, yn), of which, x1 ≤ … ≤ xn, the JoinPoint regression model can be written in log-liner form as.

$E\left[\mathrm{yi|xi}\right]=e^{\beta 0+\beta 1\mathrm{xi}+\delta 1\left(\mathrm{xi}-ϒ1\right)+\ldots \delta k\left(\mathrm{xi}-ϒk\right)+}$

where yi represents dependent variable and xi denotes independent variable for *i* = 1, 2,…, *n*; β0 represents constant parameter; β1 represents regression coefficient; δk represents the regression coefficient of the kth piecewise function. When (x_i-_ ϒ_k_) is over 1, (x_i-_ ϒ_k_) _+_ = (x_i-_ ϒ_k_), otherwise (x_i-_ ϒ_k_) _+_ = 0.

### Age-period-cohort analysis

It is known that there is a collinearity between age, period, and cohort (32). The age-period-cohort (APC) model was used to assess the temporal trends of YLDs by age, period, and cohort, which complemented standard non-parametric descriptive methods with a useful parametric framework (33).

In epidemiological terms, the age effect refers to physiological and pathological changes associated with aging. In order to assess the age effect, a longitudinal age curve and longitudinal age-specific rates were used together to adjust period deviations. The period effect are changes in disability rates caused by changes in human factors, such as advances in diagnosis technology, early detection methods, changes in disease definition and registration, treatment improvement, etc.

These human factors may affect the disease rate in different periods, resulting in a period effect. The period effect, represented in the period rate ratios, refers to changes in disease disability due to human factors, including improvements in disease diagnosis technology, screening, and early detection, changes in disease definition and registration, treatment improvement and the medical policies introduced by the Chinese government. There is a higher relative risk of disability in a period than in the reference period when rate ratios (RRs) are over 1, and a lower relative risk of disability in this period when RRs are below 1. The cohort effects refer to differences in disease mortality caused by lifestyle changes or exposure to risk factors among generations, as shown by cohort RRs (32). Net drift shows an overall log-linear trend over a calendar year and cohort, which represents an overall percentage change on an annual basis. Local drift is represented as a log-linear for each age group, based on period and birth cohorts, representing annual percentage changes (34)_._ Like previous studies (35), the data of age and period were divided into different groups with 5 years as group range. The disability and population age group started with 45–49 and completed with 90–94 and 95+. Consecutive 5-year periods were defined from 1990–1994 to 2015–2019. Consecutive 5-year cohorts were defined from 1900–1905 to 1970–1975. APC model can be written in linear regression form as follow (36):

$M=\mu +\alpha_{i}*\mathrm{age}+\beta_{j}*\mathrm{period}+\gamma_{k}*\mathrm{cohort}+\varepsilon$ .

*M* represents the death rate for Age *i* group during *j* period; αi denotes age effect of the Age *i* group; βj represents period effect of the Period *j*; γk denotes cohort effect of the NO.k (*k* = *I* + j − 1) birth cohort; μ is intercept or adjusted mean YLDs rate, and ε is the residual or a random error. The age-period-cohort web tool and the R statistical software (version 3.5.1) were used to conduct this analysis.^3^ *p* < 0.05 was considered significant (37).

## Results

### The trend in the ASDR of knee OA attributable to high BMI in China

There were 549,963.5 YLDs of knee OA attributable to high BMI in 2019 in China, with an ASDR of 25.6 per 100,000, which had increased by 460.7% since 1990 (Table 1). From 1990 to 2019, the EAPC in ASDR was 3.39 (EAPC: 3.39%; 95% CI: 3.24, 3.53%). The age-standardized YLDs rate of knee OA attributable to high BMI in China increased 3% annually, with similar trends observed in both genders (AAPC: 3.0%; 95% CI: 2.88, 3.16%). The EAPC and AAPC of China were higher than global EAPC and AAPC (EAPC of globe: 1.46%; 95% CI: 1.44, 1.48%; AAPC of globe: 1.42%; 95% CI: 1.36, 1.48%), which presents a heavier burden of knee OA attributable to high BMI in China.

**Table 1:** YLDs, ASDR, and AAPC of knee OA attributable to high BMI in 1990 and 2019 and the temporal trends from 1990 YLDs, years lived with disability; No., number; ASDR, age standardized YLDs rate; UI, uncertainty interval; EAPC, estimated annual percentage change; CI, confidential interval; AAPC, average annual percent change.

YLDs	Gender	1990		2019		1990–2019	1990–2019
Locations		YLDs cases NO. ×10^3^ (95% UI)	ASDR per 10,0000 no. (95% UI)	YLDs cases NO. ×10^3^ (95% UI)	ASDR per 10,0000 no. (95% UI)	EAPC NO. (95% UI)	AAPC NO. (95% UI)
China	Male	32655.6 (4805.9–103252.3)	7.1 (1–22.4)	189048.6 (50146.9–463413.1)	17.8 (4.7–43.9)	3.58 (3.4–3.76)	3.263 (3.093–3.432)
	Female	65426.2 (10373.3–187931.7)	14.5 (2.3–41.8)	360,915 (93113.1–869492.5)	33.1 (8.5–79.9)	3.27 (3.14–3.4)	2.908 (2.841–2.975)
	Both	98081.8 (16592.4–275584.2)	10.8 (1.8–30.4)	549963.5 (162522.4–1325608.3)	25.6 (7.5–61.6)	3.39 (3.24–3.53)	3.019 (2.875–3.164)
Globe	Male	267426.8 (82876.5–616875.3)	13.9 (4.3–32.1)	913376.3 (320535.5–2000896.1)	22.7 (8–50)	1.75 (1.73–1.77)	1.707 (1.667–1.747)
	Female	557576.9 (192828.8–1282663.6)	26.1 (9–60.2)	1664070.4 (626542.6–3679881.1)	38.1 (14.4–84.4)	1.36 (1.34–1.38)	1.312 (1.294–1.33)
	Both	825003.6 (286272.5–1856331.2)	20.5 (7.1–45.9)	2577446.7 (991632.9–5557943.7)	30.8 (11.9–66.6)	1.46 (1.44–1.48)	1.419 (1.362–1.476)
High SDI	Male	104,173 (34752.3–235842.1)	23.1 (7.7–52.3)	251672.3 (92434.9–549817.2)	31.5 (11.5–68.7)	1.04 (0.99–1.09)	1.078 (1.049–1.108)
	Female	221961.6 (78976.8–523040.9)	39.5 (14.1–92.4)	450344.2 (171740.4–1021008.5)	50.2 (19.4–113.3)	0.74 (0.68–0.79)	0.824 (0.806–0.842)
	Both	326134.6 (121743–718988.5)	32.2 (12–71.1)	702016.5 (277558.5–1,551,597)	41.2 (16.2–90.4)	0.78 (0.73–0.84)	0.854 (0.834–0.873)
High-middle SDI	Male	80346.3 (25722.1–183534.5)	16.4 (5.3–37.5)	241297.5 (84891.2–524327.7)	25.2 (8.9–55)	1.54 (1.49–1.59)	1.479 (1.436–1.522)
	Female	178,728 (64324.5–407550.7)	29.7 (10.7–67.3)	453,480 (171268.7–1004380.3)	41.4 (15.6–91)	1.2 (1.17–1.23)	1.146 (1.099–1.193)
	Both	259074.4 (95475.6–568606.4)	23.8 (8.7–52.6)	694777.5 (273856.6–1504871.1)	33.9 (13.4–73.3)	1.27 (1.23–1.3)	1.209 (1.156–1.263)
Middle SDI	Male	55340.4 (15164.7–141164.8)	10.1 (2.8–25.6)	275324.7 (95626.3–609401.1)	21.2 (7.3–46.9)	2.7 (2.64–2.76)	2.597 (2.547–2.647)
	Female	105776.8 (32886.5–248146.7)	19 (5.9–44.7)	501608.1 (183994.4–1076171.8)	36.5 (13.4–78.4)	2.46 (2.38–2.54)	2.284 (2.244–2.323)
	Both	161117.2 (49113.7–370053.7)	14.6 (4.5–33.4)	776932.8 (289722–1683491.6)	29.1 (10.8–62.6)	2.56 (2.49–2.63)	2.403 (2.329–2.477)
Low-middle SDI	Male	19710.6 (4564.4–53645.3)	6 (1.4–16.4)	110937.5 (36213.5–250659.2)	15.8 (5.1–35.9)	3.48 (3.43–3.53)	3.356 (3.244–3.468)
	Female	37295.4 (10176.4–90334.9)	11.8 (3.2–28.8)	199624.9 (68682.8–452056.2)	26.8 (9.2–60.9)	3.1 (3.04–3.17)	2.9 (2.807–2.994)
	Both	57,006 (14942–142550.8)	8.9 (2.3–22.3)	310562.4 (110675.6–685762.8)	21.5 (7.6–47.6)	3.29 (3.22–3.35)	3.11 (3.062–3.157)
Low SDI	Male	7690.5 (1672.6–21070.8)	5.9 (1.3–16.1)	33597.3 (10277–77863.9)	11.9 (3.6–27.6)	2.58 (2.47–2.69)	2.477 (2.416–2.537)
	Female	13476.5 (3450.6–34,217)	10.7 (2.7–27.3)	58030.2 (19308.8–137554.6)	20.2 (6.7–48.1)	2.37 (2.26–2.47)	2.232 (2.189–2.274)
	Both	21167.1 (5420.7–53253.6)	8.2 (2.1–20.8)	91627.5 (30790.6–209079.2)	16.1 (5.3–37)	2.48 (2.37–2.58)	2.351 (2.315–2.386)

### Knee OA burden attributable to high BMI by ages and genders in China

**Figure 1:**
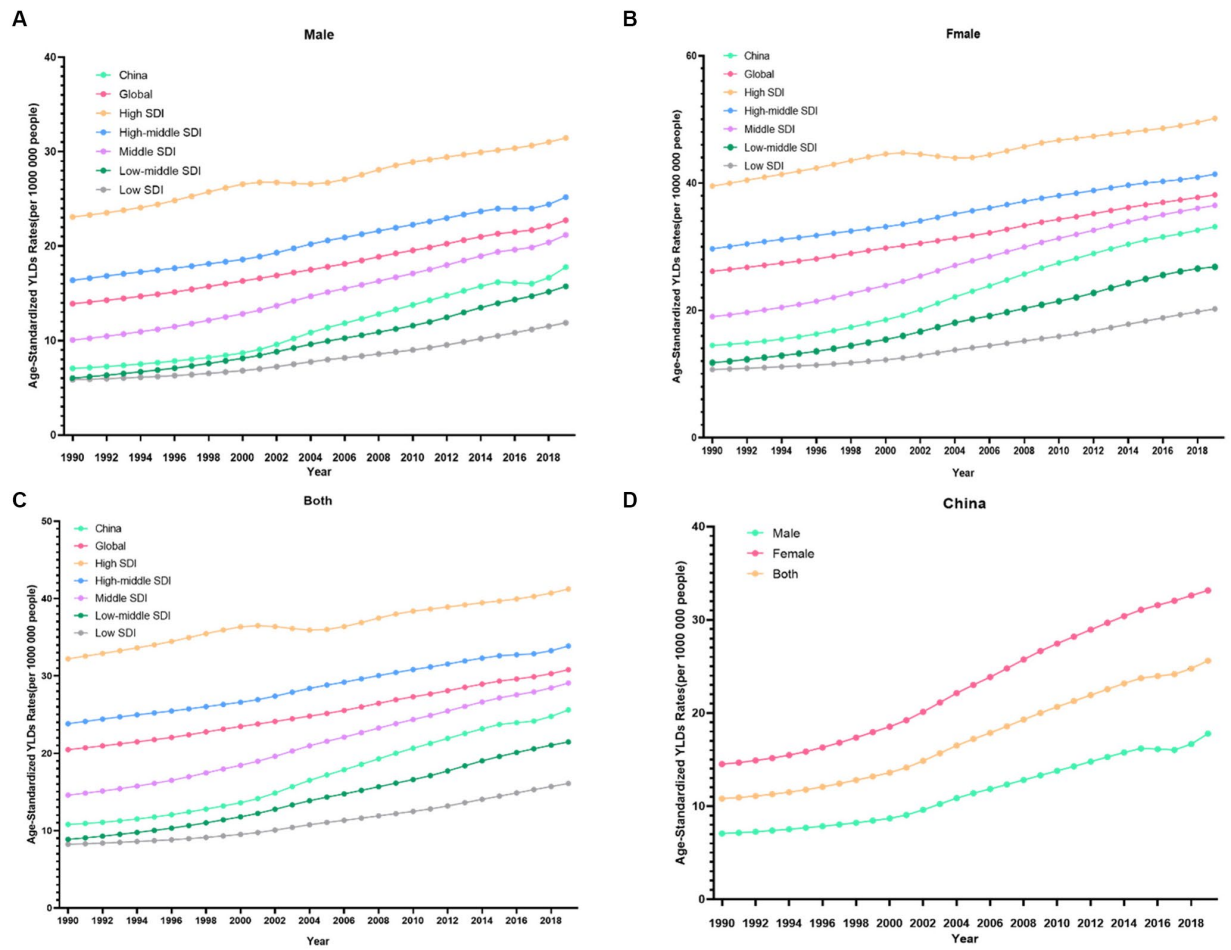
Changes in the ASDR of knee OA attributable to high body mass index globally and in different socio-economic index regions from 1990 to 2019. **(A)** Male. **(B)** Female. **(C)** Both. **(D)** China.

**Figure 2:**
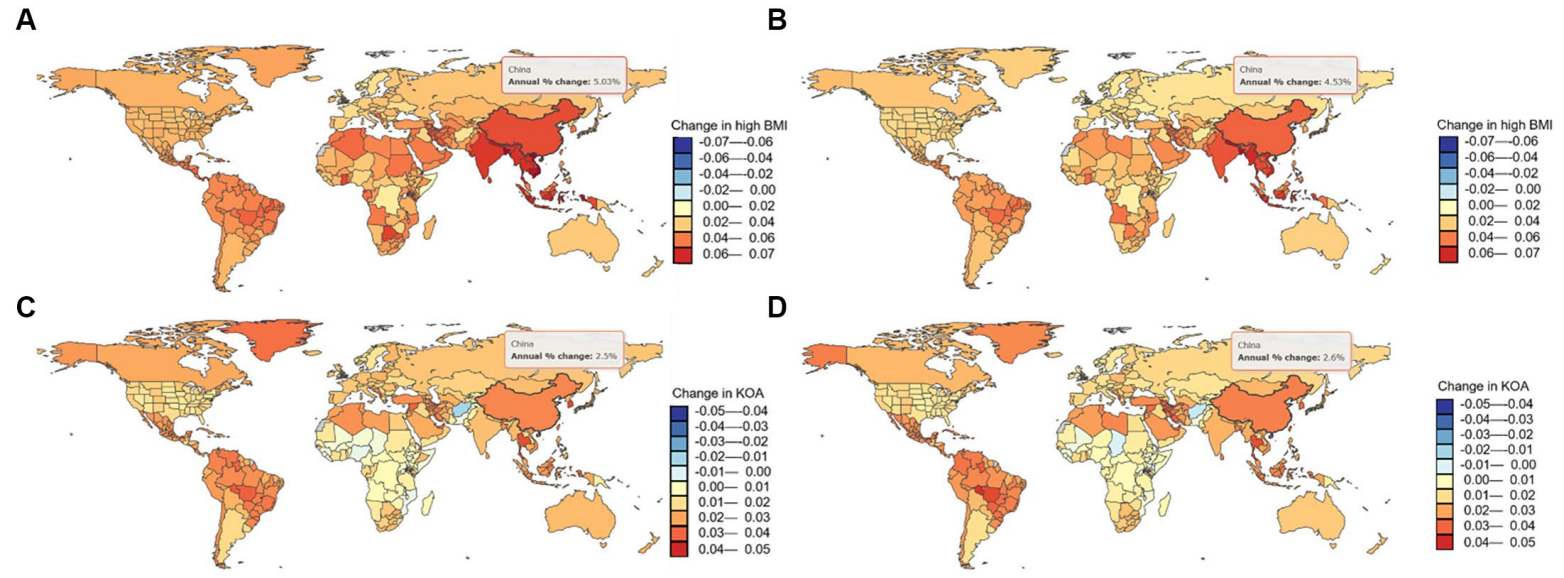
The number of YLDs attributable to high BMI in China changed between 1990 and 2019. **(A)** Males; and **(B)** Females; The number of YLDs of knee OA in China changed between 1990 and 2019 **(C)** Males; **(D)** Females.

From 1990 to 2019, the trends in the ASDR of knee OA due to high BMI by genders in China are shown in Figure 1D. Both men and women showed significant increasing trends of suffering from knee OA attributable to high BMI in China over the past 30 years. Although the trend was similar between men and women, women showed a rapider increasing trend. In the Joinpoint regression analyses, this increasing trend was more pronounced among men (AAPC: 3.26%; 95% CI: 3.10, 3.43%) and women (AAPC: 2.91%; 95% CI: 2.84, 2.98%) in China than that of the Global (AAPC: 1.71%; 95% CI: 1.67, 1.75%; 1.31%; 95% CI: 1.29, 1.33%) (Table 1), and the EAPC of knee OA increased more among males (EAPC: 3.58%; 95% CI: 3.40, 3.76%) than females (EAPC 3.27%; 95% CI: 3.14, 3.40%) (Figure 2). In China, women presented higher YLD rates for knee OA associated with high BMI than men of all ages (ASDR: 33.1 vs. 17.8) from 1990 to 2019, while men’s changes were more significant (AAPC: 3.263 vs. 2.908) (Table 1).

**Figure 3:**
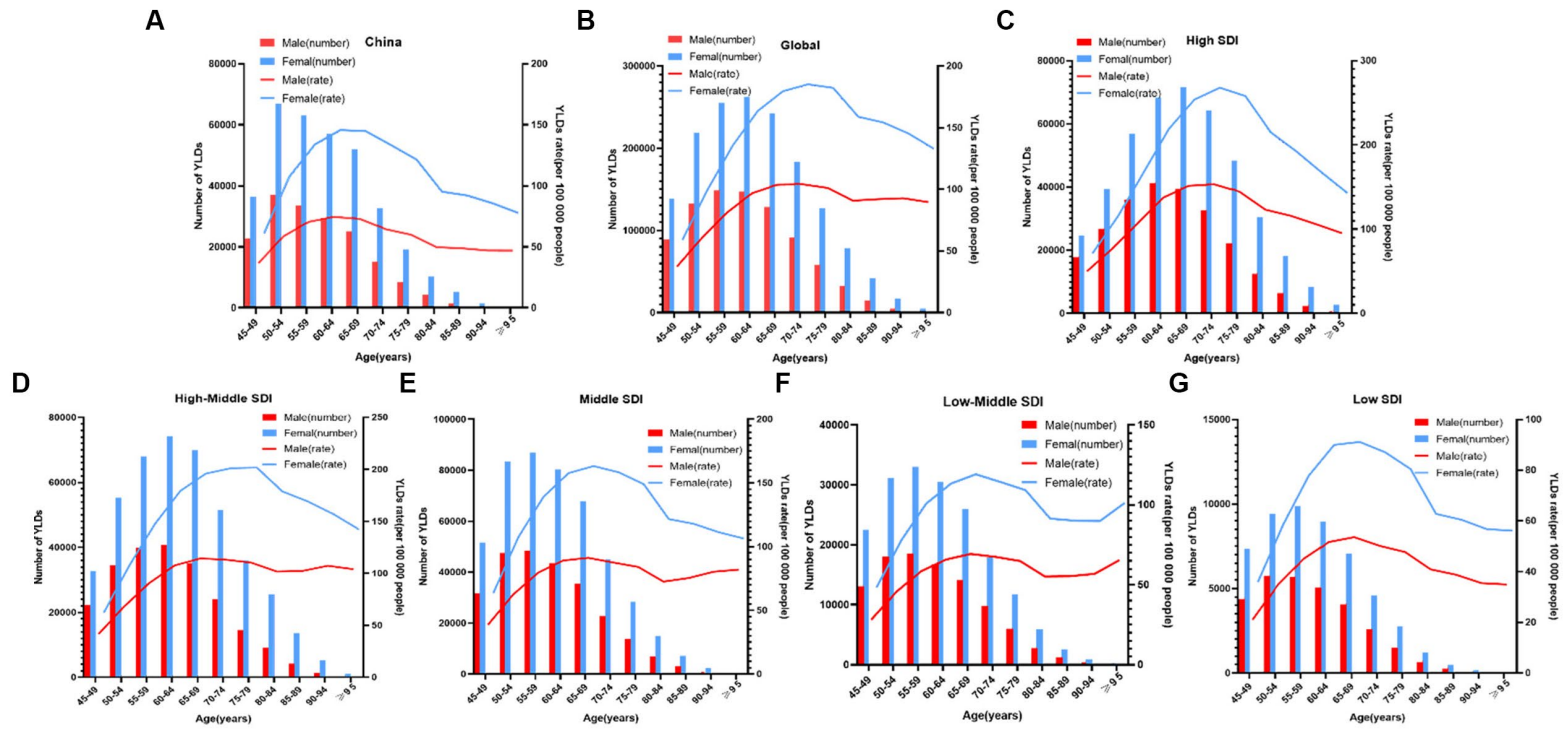
Age-specific numbers and rates of years lived with disability (YLDs) of knee OA attributable to high body mass index by age and gender, in 2019. **(A)** China; and **(B)** Globe; and **(C)** High SDI; and **(D)** High-Middle SDI; and **(E)** Middle SDI; and **(F)** Low-Middle SDI; and **(G)** Low SDI.

The YLDs rate and the number of patients with knee OA due to high BMI by age in China between 1990 and 2019 were shown in Figure 3A. Up to 2019, of all the age groups, YLDs for knee OA due to high BMI first increased and then decreased after 50–54 years for both men and women, the rate of YLDs of knee OA due to high BMI first increased and then decreased after 60–69 years for both men and women in China.

### The age-period-cohort analysis of the YLDs rate of knee OA attributable to high BMI in China

**Figure 4:**
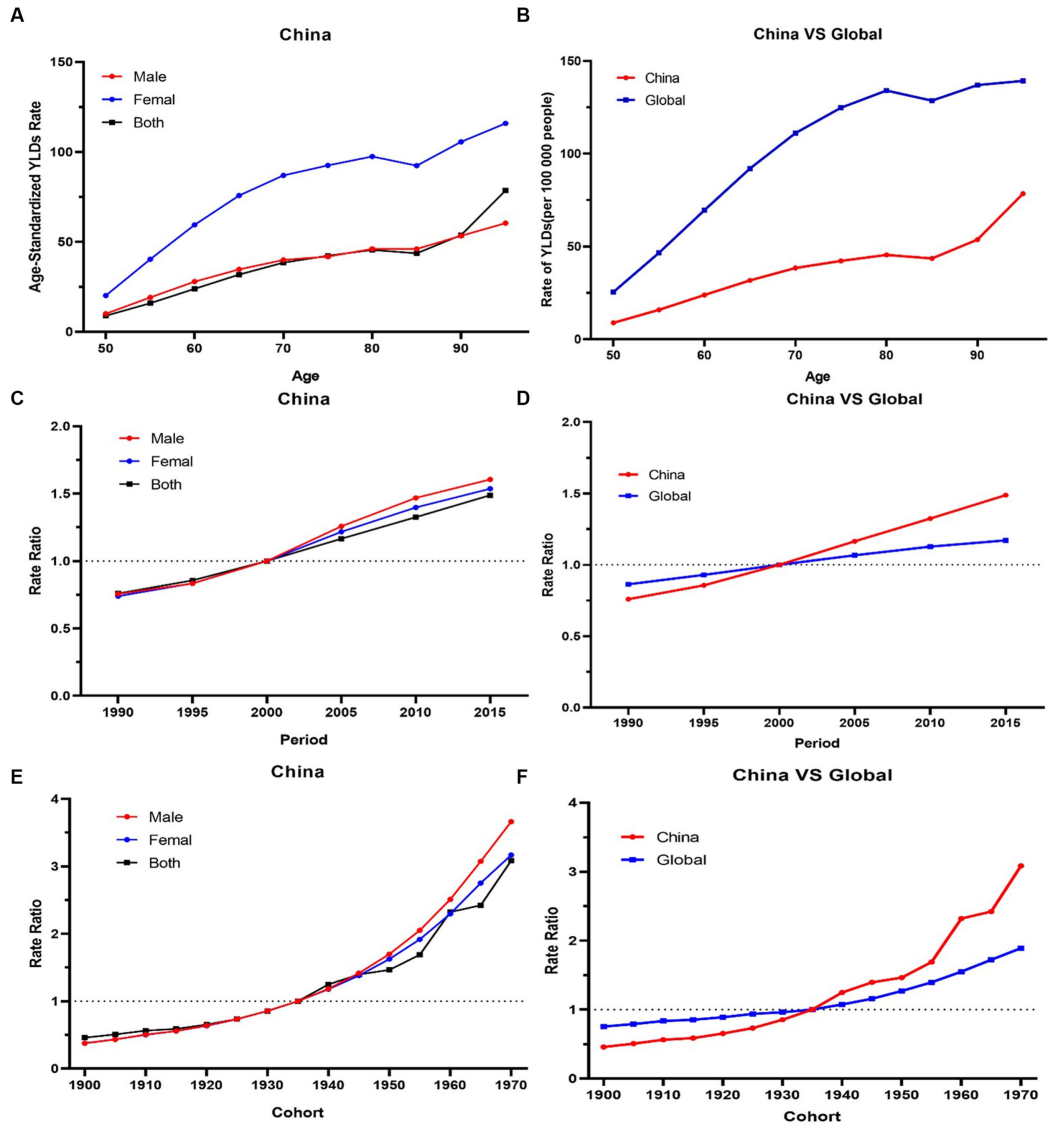
**(A,B)** The longitudinal age curves of knee OA by gender in high BMI in China and globe. **(C,D)** The period RRs of knee OA by gender in high BMI in China and globe. **(E,F)** The cohort RRs of knee OA by gender in in high BMI in China and globe.

For the same birth cohort, the YLDs rate of knee OA attributable to high BMI increased with age. The YLDs rate of knee OA among females was higher than that among males. The YLDs rate of knee OA attributable to high BMI had increasing trends with the increase of age, particularly, the 50–80 age groups increasing rapidly (Figure 4A).

The period RRs of knee OA attributable to high BMI showed an increasing trend from 1990 to 2019. The upward trend among females was similar with that among males before 2000, and the upward trend among males was steeper than that among females from 2000 to 2019 (Figure 4C).

Cohort RRs of knee OA attributable to high BMI had an obvious increasing trend among those born between 1900 to 1970. Similar trends were observed among both males and females (Figure 4E).

The overall net drift values of knee OA attributable to high BMI were above 1. Compared with the globe, the net drift values of knee OA attributable to high BMI in China was higher (China 2.8% vs. globe 1.2%, *p* < 0.001). In terms of gender, in China, the net drift value of knee OA attributable to high BMI for males was 3.3%, and for females, it was 3.1% (*p* < 0.001). Males had higher net drift values compared with females. In 52.5–77.5 age group, the local drift values of knee OA decreased with age increasing in China.

### Knee OA burden attributable to high BMI in globe and regions with different SDI

Globally, the YLDs increased by 2.12 times, from 825003.6 in 1990 to 2577446.7 in 2019, with an ASDR of 30.8 per 100,000 (Table 1). The age-standardized EAPC was 1.46 from 1990 to 2019. The trends showed a steady and large increase in men and women globally from 1990 to 2019. Global YLDs rate was higher than that of China, and YLDs rates of both increased with age attributable to high BMI under 80 and slightly decreased with age exceeding 80, they trended upwards rapidly after the 80–85 age groups (Figure 4B). The global period RRs of knee OA attributable to high BMI was lower than that of China after 2000 (Figure 4D), the global cohort RRs was lower than China after 1935 (Figure 4F). In 82.5–92.5 age group, the local drift values of knee OA increased with age.

Figure 3 showed the distribution discrepancy among different age groups in countries with different SDI levels in 2019. Both males and females aged 70–74 had the highest YLDs rate in higher-SDI regions, while the YLDs rate peaked in the 65–69 age group in middle and low-middle and low-SDI regions (Figures 3C–G). For areas with different SDI levels, the YLD rate was higher in high-SDI areas compared with low-SDI areas in the same age group. From 1990 to 2019 (Figure 1), in regions with the 5 different SDI levels, the highest ASDR of high BMI-related knee OA were observed in high-SDI regions (41.2 per 100,000), followed by regions with high-middle SDI (33.9 per 100,000) and middle-SDI (29.1 per 100,000), whereas the lowest ASDR were regions with low SDI (16.1 per 100,000), followed by low-middle SDI (21.5 per 100,000) (Table 1). The EAPC of ASDR in high SDI, high-middle SDI, middle SDI, low-middle SDI, low-middle were 0.78, 1.27, 2.56, 3.29, 2.48 respectively, which means the ASDR of knee OA attributable to high BMI in the above five regions increased in the past 30 years (Table 1).

The results of the APC model regarding the age, period, and cohort effect were shown in Supplementary Tables S1–S9 in detail.

## Discussion

OA is a multifactorial chronic disease that affects multiple joints throughout hand, hip, knee, and foot (38). It’s estimated that 240 million individuals worldwide have symptomatic OA (2). Knee joint is the most common site of osteoarthritis (39, 40), which at end stage may lead to joint replacement. Results show that disease burden of knee OA in China is higher than the global burden as well as that in other regions with high SDI. The AAPC in China was 3.54 times that of the regions with high SDI, and 2.13 times that of the world, which suggests that China still needs to make efforts in the prevention and control of knee OA.

Studies (41–44) have proved that higher BMI is positively correlated with greater lower limb joint load, obesity can place an extra strain on joints and change the composition, structure and mechanical properties of cartilage to exert pathogenic effect. Moreover, during disease progression, to avoid weight-bearing pain, patients will take slight knee hyperextension maneuver to reduce joint surface pressure, which will lead to uneven distribution of intra joint force of the knee (45), further injuring the knee.

In this article, the relationship between high BMI and knee OA onset is extensively discussed. Based on Joinpoint regression and age-period-cohort models, this study systematically estimated the change of the YLDs for knee OA attributable to high BMI in China and globally from 1990 to 2019. On the basis of the latest literature (3, 46, 47) available for knee OA, the disease burden associated with high BMI in China was quantified, and the present study, found that YLDs for knee OA attributable to high BMI has substantially increased over the past 30 years. The ASDR reached 25.6 (95% UI: 7.5, 61.6) per 100,000 in 2019, which increased by 137.0% compared with 1990. The YLDs for knee OA associated with high BMI accounted for 16.7% in 2019, compared with the all-time YLDs for knee OA shown at the following link: https://vizhub.healthdata.org/gbd-results/. In terms of the development trend, there is a similar increase trend in YLDS in China and around the world between 1990 and 2019, which indicates that the severe disease burden China faces is also an urgent global problem.

Then, the effects of age on the epidemiological changes in knee OA attributable to high BMI in China were analyzed further. Results showed that there was a significant increase in ASDR for knee OA due to high BMI among the 47.5–92.5 age group, while the values of those younger than 47.5 were lower. There is evidence that healthcare costs of knee OA attributable to high BMI are increasing faster in older individuals than in young and middle-aged people (47). On the one hand, this is because as obesity rates increase in the elderly, hypertrophic fat cells accumulate in joints and contribute to local inflammation (48). On the other hand, when cells divide, repeated sequences are lost, resulting in shortening of telomeres (49). Chondrocytes will not divide in general adulthood. Nevertheless, chondrocyte telomeres have been shown to shorten in elderly people. This may be due to chondrocytes more susceptible to environmental pressures in that age group (50). Similar epidemiological changes in knee OA attributable to high BMI were found in terms of period and cohort. Results showed that the cohort RRs of knee OA attributable to high BMI trend increased among those born from 1900 to 1970, indicating middle-aged and elderly people may have a higher risk to suffer from knee OA. The period RRs of knee OA attributable to high BMI trended upward, indicating that although the government has attempted to improve the state of high BMI in China, it must formulate an effective policy to reduce the burden caused by knee OA due to high BMI among elderly people.

Extensive evidence confirms gender differences in prevalence in OA (51, 52). Women are more susceptible to hands, feet, and knee OA than men, but less prone to spondylarthritis (53). In this study, the ASDR attributable to high BMI for both genders showed similar trends, but women were more significantly burdened by knee OA than men, which was consistent with previous studies, meaning that women needed more treatment than men (42). There may be several reasons: (1) Women may experience stronger inflammation during fat accumulation, and estrogen deficiency after menopause increases free fatty acid levels, which will exacerbate the pain caused by knee OA (43). (2) Subcutaneous fat expresses more estrogen receptors than visceral fat, and visceral fat expresses more androgen receptors than subcutaneous fat. Besides, when involved in inflammatory responses, subcutaneous abdominal fat affects knee joint biomechanics as well (44). As a result of having more subcutaneous fat, women’s knees are more loaded and experience higher shock (45). (3) Women may be more sensitive to changes occurring in their physical health and may go to the hospital more frequently for check-ups, making the change in joint function more apparent.

In addition, the prevalence of knee OA in regions with different SDI around the world was also documented. Osteoarthritis of the knee appears to have a higher incidence in developed countries, being one of the top 10 disabilities among older people in developed countries (54). The prevalence of knee OA among people over 45 years old in developed countries is 19.2%, and prevalence among people over 80 years old is 43.7% (54). In this study, burden of knee OA was more associated with high BMI in countries with high SDI than it was in countries with low SDI. In 2019, the ASDR of knee OA attributable to high BMI in high-SDI regions was 2.56 times that in regions with low SDI, and 1.41 times that in the middle-SDI regions. This may be due to poor economic condition in regions with low SDI, where to get adequate food is still a problem. Meanwhile, more people engage in physical activities which require a high level of energy expenditure, so the risk of knee OA is relatively low, and the disease burden of knee OA is moderate (55). In addition, lower-income people consumed more fruits and vegetables, while higher-income people consumed more fat, salt, and processed foods, which are more likely to make people obese. As a developing country, China’s ASDR level is between middle SDI and low-middle SDI, and the ASDR of knee OA disability caused by high BMI will increase year by year.

The advantage of this study is that it complemented shortcomings of previous studies, but it has several limitations. First, the data are only from the Global Burden of Disease. Actually, relevant knee OA information from other databases should be collected and analyzed in future studies. Second, data of other countries are lacked, and focus was primarily concentrated on China in this study. As a result, the conclusions of the study are regional, and the generalizability of the conclusions of the study needs to be improved. Third, data only involve knee OA, so a whole scope of the entire burden of OA cannot be obtained. It is expected that future GBD studies will include OA of other joints, such as the hip, hands, and further clarify the true burden of OA. Finally, the risk factors of knee OA in GBD are not comprehensive enough, and this study lacks date on other external factors for OA, such as changes in physical activity levels, diet patterns, or advancements in medical treatments.

In summary, the burden of knee OA in China increased between 1990 and 2019. As one of the major risk factors of knee OA, high BMI also contributes to many other chronic diseases, such as diabetes (56). Although gender and age are innate factors, understanding knee OA epidemic patterns can reduce the disease burden. With regard to formulating policies, the government can provide targeted guidance, such as strengthening nursing care, organizing regular examinations, and encouraging women and the elderly to participate in more physical activities (10, 57–59), all of which is beneficial to reducing the disease burden of susceptible groups (8). To stay health is not only to prevent death，but also to reduce the burden of disease from chronic disease. There should be resources available for preventing, treating, and ameliorating non-fatal sequelae of disease. At present, the management model of chronic diseases of knee OA in China is yet being explored. In view of this, knee OA should be considered as part of the chronic disease management system to delay the disease process and reduce the disability rate as well as improve the quality of life through scientific management of patients.

## Conclusion

To conclude, knee OA attributable to high BMI is more harmful to Chinese women and the elderly from 1990 to 2019. To reduce the burden of knee OA attributable to high BMI, the Chinese government should make effective public health policies and take timely measures to protect specific populations with high BMI. Based on findings in this study, the government can take targeted actions to manage knee OA and high BMI in different regions in the future.

## Data availability statement

The original contributions presented in the study are included in the article/Supplementary material, further inquiries can be directed to the corresponding authors.

## Ethics statement

Ethical approval was not required for the study involving humans in accordance with the local legislation and institutional requirements. Written informed consent to participate in this study was not required from the participants or the participants’ legal guardians/next of kin in accordance with the national legislation and the institutional requirements. The manuscript presents research on animals that do not require ethical approval for their study. Written informed consent was obtained from the individual(s) for the publication of any potentially identifiable images or data included in this article.

## Author contributions

MS and HC: conceptualization and methodology. AZ and JL: data extraction. WW and WH: investigation. JY: project administration. GW and JL: resources. MS and WW: software. MS and QY: writing—original draft. QY and JY: writing—review and editing. All authors contributed to the article and approved the submitted version.

## Funding

This work was supported by Scientific Research Project of National Natural Youth Science Foundation of China (Grant number: 82104964) and Scientific Research Project of National Natural Science Foundation of China (Grant numbers: 81973950). The funding bodies had no role in the design of the study, the collection, analysis, or interpretation of the data, or writing the manuscript.

## Conflict of interest

The authors declare that the research was conducted in the absence of any commercial or financial relationships that could be construed as a potential conflict of interest.

## Publisher’s note

All claims expressed in this article are solely those of the authors and do not necessarily represent those of their affiliated organizations, or those of the publisher, the editors and the reviewers. Any product that may be evaluated in this article, or claim that may be made by its manufacturer, is not guaranteed or endorsed by the publisher.

## Footnotes

^1^http://ghdx.healthdata.org/gbd-results-tool

^2^https://vizhub.healthdata.org/gbd-results/

^3^https://analysistools.cancer.gov/apc/

## Abbreviations

Knee OA: Knee osteoarthritis
YLDs: Years lived with disability
DALYs: Disability-adjusted life-years
GBD: Global Burden of Disease
APC: Age-period-cohort
AAPC: Average annual percent change
ASDR: age-standardized disability-adjusted life year rate
SDI: Socio-demographic index
BMI: Body mass index
RRs: Rate ratios
UIs: Uncertainty intervals
CIs: Confidence intervals
