# Supplementary material for: A comparison of the burden of knee osteoarthritis attributable to high body mass index in China and globally from 1990 to 2019
Source: Front Med (Lausanne). 2023 Aug 23;10:1200294. doi: 10.3389/fmed.2023.1200294 (PMC10481341; doi:10.3389/fmed.2023.1200294)
Supplement: Supplementary file 1 [file Table_1.DOCX]

| Age | Rate | CILo | CIHi |
| --- | --- | --- | --- |
| 47.5 | 0.024 | 0.0133 | 0.0431 |
| 52.5 | 0.0248 | 0.0144 | 0.0429 |
| 57.5 | 0.0261 | 0.0161 | 0.0422 |
| 62.5 | 0.0276 | 0.0174 | 0.0437 |
| 67.5 | 0.0305 | 0.0195 | 0.0477 |
| 72.5 | 0.0359 | 0.0229 | 0.0562 |
| 77.5 | 0.0487 | 0.0308 | 0.0771 |
| 82.5 | 0.0826 | 0.0511 | 0.1335 |
| 87.5 | 0.2192 | 0.127 | 0.3782 |
| 92.5 | 1.3306 | 0.7398 | 2.3933 |

Table 1. Long age of male

| Age | Rate | CILo | CIHi |
| --- | --- | --- | --- |
| 47.5 | 0.0273 | 0.0152 | 0.0491 |
| 52.5 | 0.0279 | 0.0162 | 0.0482 |
| 57.5 | 0.0288 | 0.0178 | 0.0466 |
| 62.5 | 0.0299 | 0.0189 | 0.0473 |
| 67.5 | 0.0318 | 0.0203 | 0.0498 |
| 72.5 | 0.0356 | 0.0228 | 0.0558 |
| 77.5 | 0.0439 | 0.0277 | 0.0694 |
| 82.5 | 0.0622 | 0.0385 | 0.1005 |
| 87.5 | 0.1153 | 0.0668 | 0.1989 |
| 92.5 | 0.3163 | 0.1759 | 0.5689 |

Table 2. Long age of female

| Age | Rate | CILo | CIHi |
| --- | --- | --- | --- |
| 47.5 | 0.0128 | 0.0071 | 0.023 |
| 52.5 | 0.0132 | 0.0076 | 0.0227 |
| 57.5 | 0.0137 | 0.0085 | 0.0222 |
| 62.5 | 0.0143 | 0.0091 | 0.0227 |
| 67.5 | 0.0156 | 0.01 | 0.0244 |
| 72.5 | 0.0179 | 0.0115 | 0.0281 |
| 77.5 | 0.0231 | 0.0146 | 0.0366 |
| 82.5 | 0.0353 | 0.0218 | 0.057 |
| 87.5 | 0.0744 | 0.0431 | 0.1284 |
| 92.5 | 0.2477 | 0.1377 | 0.4455 |

Table 3. Long age of both

| Period | Rate Ratio | CILo | CIHi |
| --- | --- | --- | --- |
| 1992.5 | 1.3684 | 0.9421 | 1.9875 |
| 1997.5 | 1.1814 | 0.8211 | 1.6997 |
| 2002.5 | 1 | 1 | 1 |
| 2007.5 | 0.833 | 0.5796 | 1.1973 |
| 2012.5 | 0.6948 | 0.4819 | 1.0018 |
| 2017.5 | 0.5568 | 0.3837 | 0.808 |

Table 4. PeriodRR of male

| Period | Rate Ratio | CILo | CIHi |
| --- | --- | --- | --- |
| 1992.5 | 1.3143 | 0.9049 | 1.9089 |
| 1997.5 | 1.166 | 0.8105 | 1.6776 |
| 2002.5 | 1 | 1 | 1 |
| 2007.5 | 0.8347 | 0.5808 | 1.1997 |
| 2012.5 | 0.6801 | 0.4717 | 0.9807 |
| 2017.5 | 0.5411 | 0.3728 | 0.7852 |

Table 5. PeriodRR of female

| Period | Rate Ratio | CILo | CIHi |
| --- | --- | --- | --- |
| 1992.5 | 1.3294 | 0.9153 | 1.9308 |
| 1997.5 | 1.1704 | 0.8135 | 1.6838 |
| 2002.5 | 1 | 1 | 1 |
| 2007.5 | 0.8331 | 0.5796 | 1.1973 |
| 2012.5 | 0.6817 | 0.4728 | 0.9829 |
| 2017.5 | 0.5435 | 0.3745 | 0.7887 |

Table 6. PeriodRR of both

| Cohort | Rate Ratio | CILo | CIHi |
| --- | --- | --- | --- |
| 1900 | 5.6509 | 2.0437 | 15.6253 |
| 1905 | 4.0358 | 1.8693 | 8.7133 |
| 1910 | 3.0717 | 1.6001 | 5.8966 |
| 1915 | 2.3619 | 1.3208 | 4.2236 |
| 1920 | 1.9272 | 1.1324 | 3.2796 |
| 1925 | 1.4831 | 0.9051 | 2.4303 |
| 1930 | 1.1631 | 0.7245 | 1.8674 |
| 1935 | 1 | 1 | 1 |
| 1940 | 0.9556 | 0.5952 | 1.5341 |
| 1945 | 0.8329 | 0.5083 | 1.3648 |
| 1950 | 0.6369 | 0.3743 | 1.0839 |
| 1955 | 0.5378 | 0.3007 | 0.9617 |
| 1960 | 0.5503 | 0.2867 | 1.0564 |
| 1965 | 0.4106 | 0.1902 | 0.8865 |
| 1970 | 0.4058 | 0.1468 | 1.1222 |

Table 7. CohortRR of male

| Cohort | Rate Ratio | CILo | CIHi |
| --- | --- | --- | --- |
| 1900 | 5.8194 | 2.1046 | 16.0911 |
| 1905 | 3.9057 | 1.809 | 8.4324 |
| 1910 | 2.9042 | 1.5128 | 5.575 |
| 1915 | 2.1186 | 1.1847 | 3.7885 |
| 1920 | 1.679 | 0.9866 | 2.8573 |
| 1925 | 1.3508 | 0.8244 | 2.2135 |
| 1930 | 1.1454 | 0.7134 | 1.8389 |
| 1935 | 1 | 1 | 1 |
| 1940 | 0.9543 | 0.5944 | 1.532 |
| 1945 | 0.806 | 0.4919 | 1.3207 |
| 1950 | 0.5982 | 0.3515 | 1.0181 |
| 1955 | 0.4978 | 0.2784 | 0.8901 |
| 1960 | 0.5102 | 0.2658 | 0.9794 |
| 1965 | 0.3743 | 0.1734 | 0.8081 |
| 1970 | 0.3752 | 0.1357 | 1.0374 |

Table 8. CohortRR of female

| Cohort | Rate Ratio | CILo | CIHi |
| --- | --- | --- | --- |
| 1900 | 5.9637 | 2.1568 | 16.4901 |
| 1905 | 4.0563 | 1.8788 | 8.7576 |
| 1910 | 3.0321 | 1.5795 | 5.8205 |
| 1915 | 2.239 | 1.2521 | 4.0038 |
| 1920 | 1.7757 | 1.0435 | 3.0219 |
| 1925 | 1.4031 | 0.8563 | 2.2991 |
| 1930 | 1.1569 | 0.7206 | 1.8573 |
| 1935 | 1 | 1 | 1 |
| 1940 | 0.9536 | 0.594 | 1.5309 |
| 1945 | 0.8186 | 0.4995 | 1.3413 |
| 1950 | 0.6165 | 0.3623 | 1.0492 |
| 1955 | 0.517 | 0.2891 | 0.9246 |
| 1960 | 0.5303 | 0.2762 | 1.018 |
| 1965 | 0.3935 | 0.1822 | 0.8495 |
| 1970 | 0.3919 | 0.1417 | 1.0838 |

Table 9. CohortRR of both
